# Supplementary figures and images for: Physics-based modeling provides predictive understanding of selectively promiscuous substrate binding by Hsp70 chaperones
Source: PLoS Comput Biol. 2021 Nov 4;17(11):e1009567. doi: 10.1371/journal.pcbi.1009567 (PMC8604352; doi:10.1371/journal.pcbi.1009567)

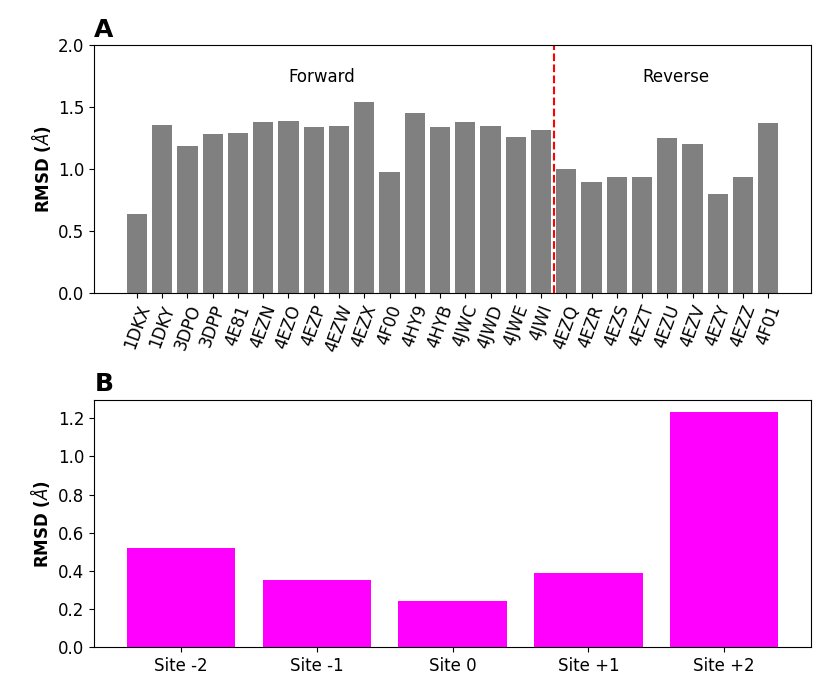

Supplement: S1 Fig — (A) RMSD of βSBD backbone atoms. (B) RMSD of the backbone of residues of substrates without prolines by site (1DKY, 4EZW, 4EZX). For both figures, the reference structure is 1DKZ. All structures were aligned to 1DKZ based on residues 393–503 (βSBD). (TIF) [file pcbi.1009567.s008.tif]

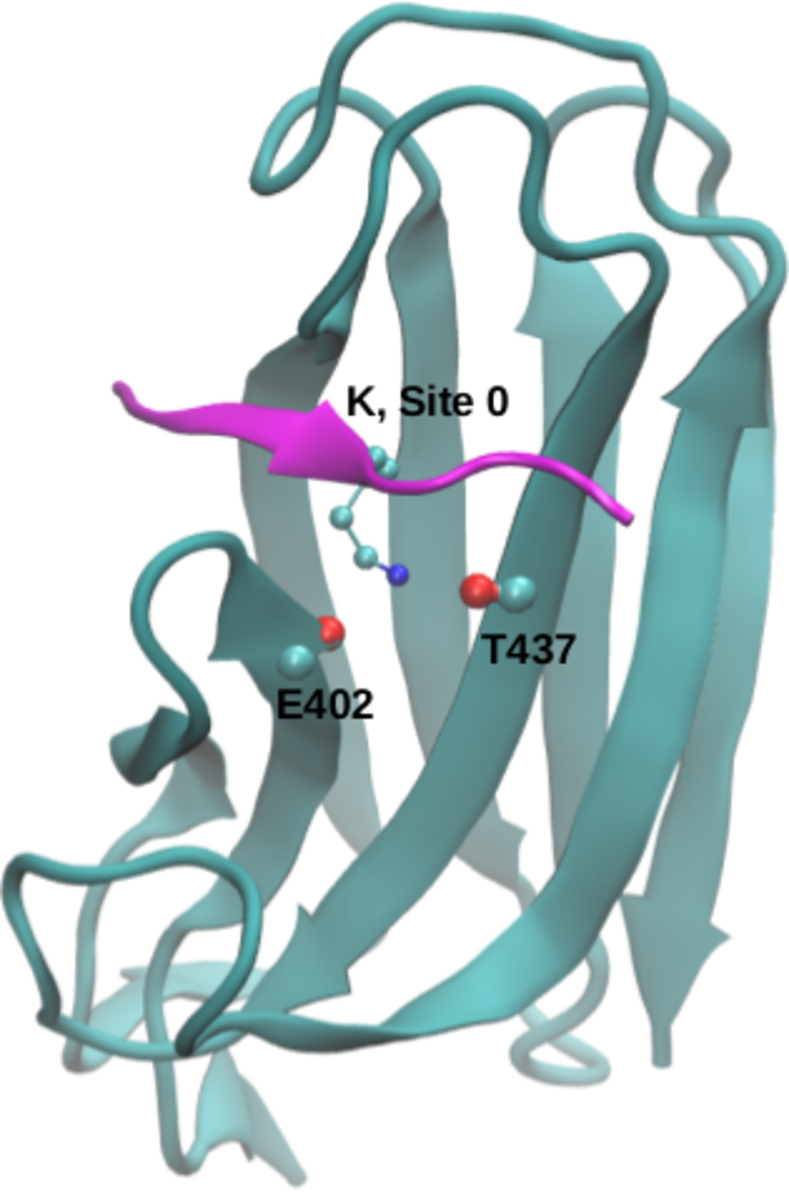

Supplement: S2 Fig — The two βSBD residues E402 and T437 have solvent exposed carbonyls which can potentially satisfy and stabilize charged and polar residues buried in site 0, illustrated here with L. This is a snapshot from an MD simulation. (TIF) [file pcbi.1009567.s009.tif]

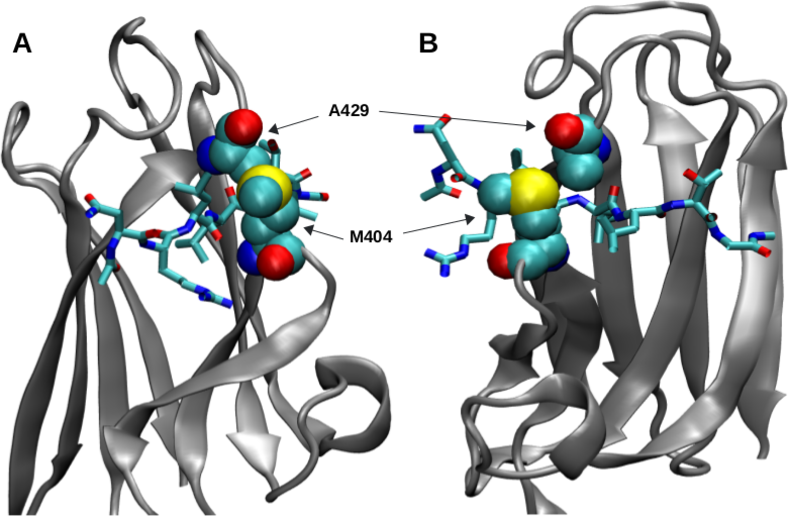

Supplement: S3 Fig — (A) The interaction between the bridging M404 and A429, which together partially cover site 0, viewed from the site -1 of the complex. (B) The same as (A) but viewed from the site +1 side. The structure shown is PDB ID: 1DKZ. (TIF) [file pcbi.1009567.s010.tif]

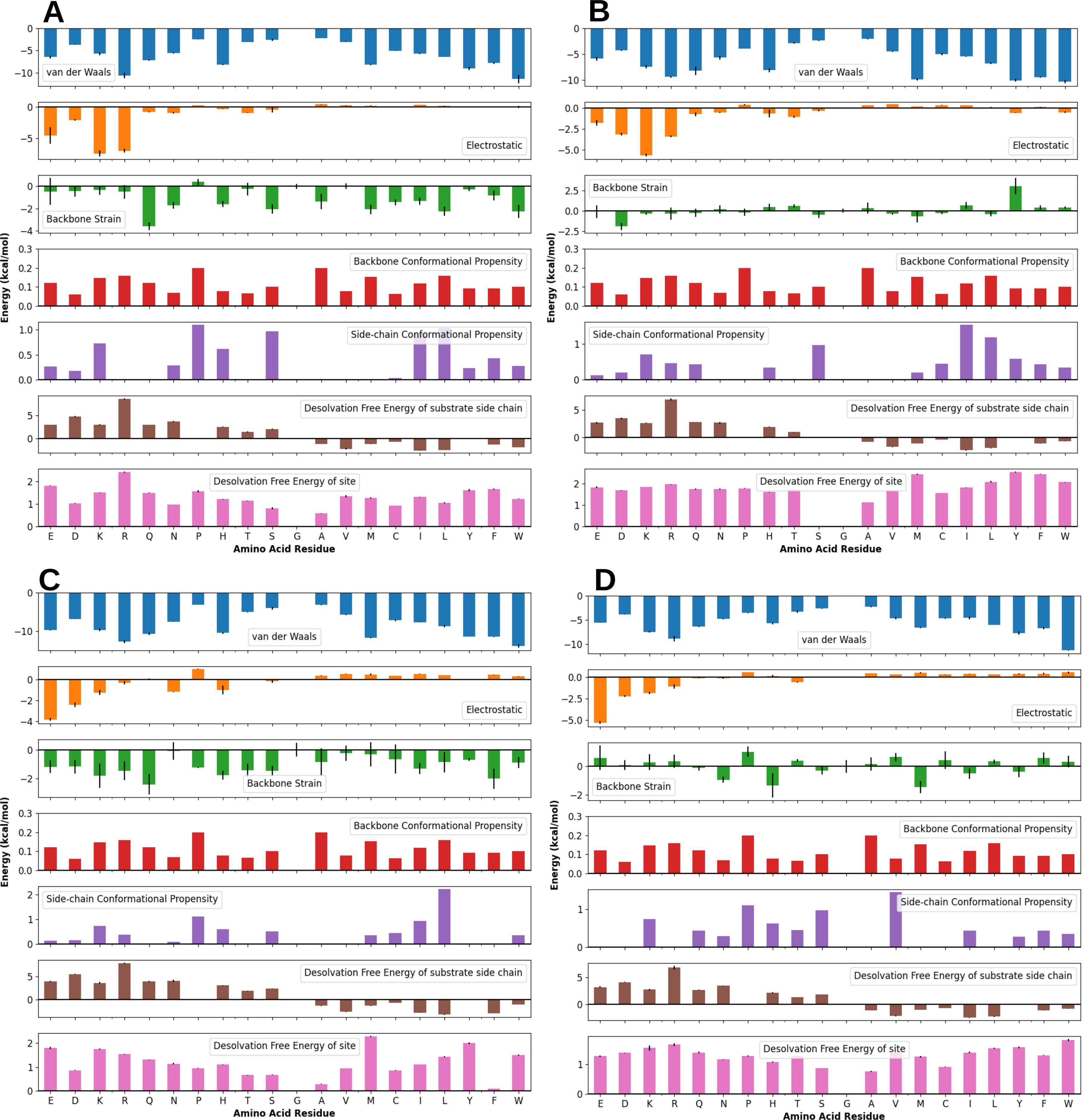

Supplement: S4 Fig — (A) site +2, (B) site +1, (C) site -1, and (D) site -2. Site 0 is included in the main text (Fig 4). (TIF) [file pcbi.1009567.s011.tif]

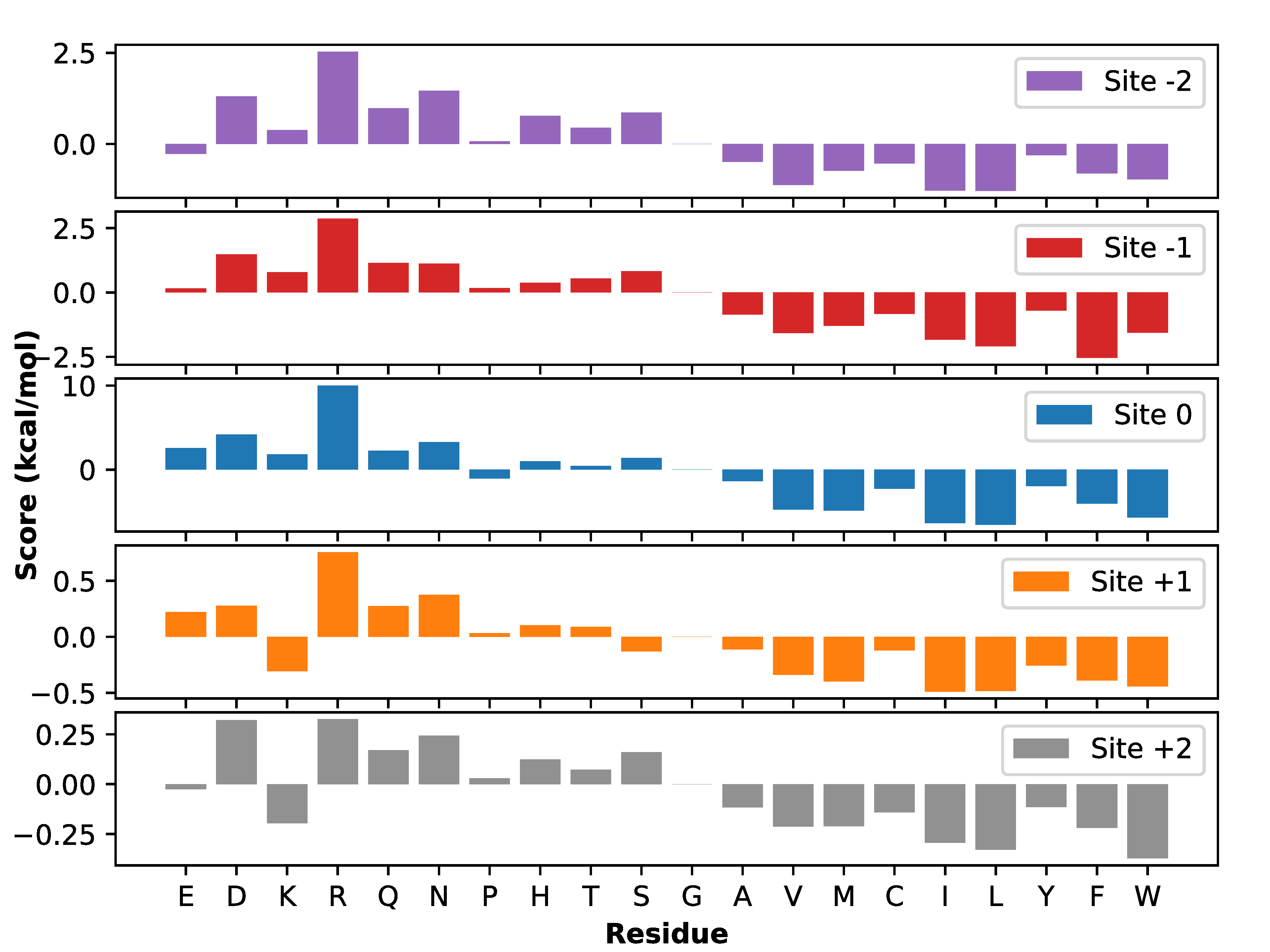

Supplement: S5 Fig — These scores are derived by multiplying the energy terms in Figs 4 and S4 by the weights in Table 1. The values from this figure are also given in S3 Table. (TIF) [file pcbi.1009567.s012.tif]

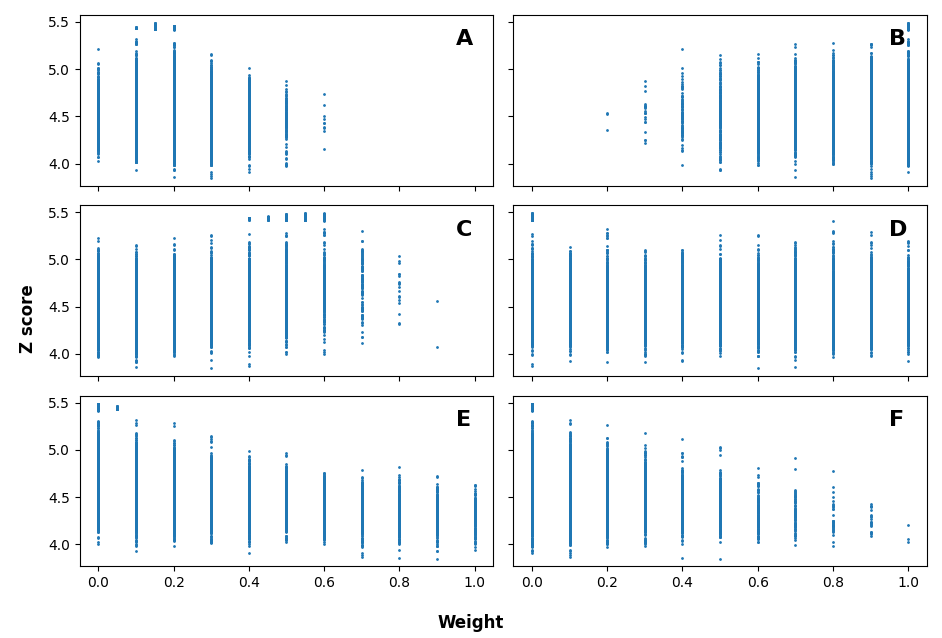

Supplement: S6 Fig — Top 1000 Z-scores vs various weights from all Monte Carlo runs (see Methods). The weights correspond to (A) vdW interaction energy, (B) Electrostatic interaction energy, (C) Backbone strain, (D) Desolvation FE of the sidechain, (E) Backbone conformational propensity, and (F) Sidechain conformational propensity. (TIF) [file pcbi.1009567.s013.tif]

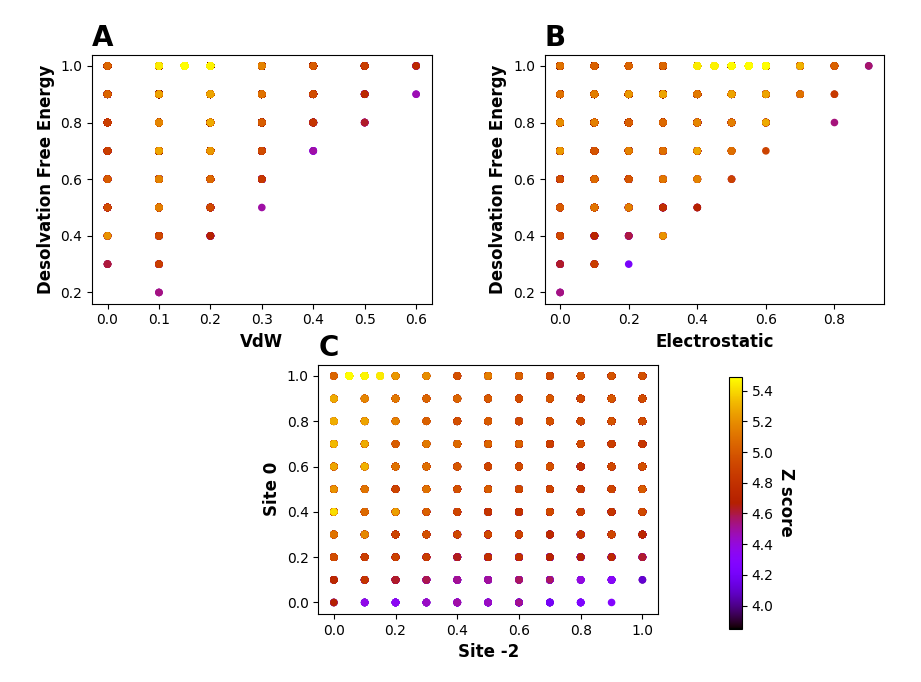

Supplement: S7 Fig — The plots show two weights as a function of Z-score along the color axis for the same top 1000 results from all Monte Carlo runs and additional grid searches as discussed above. The panels show the weight combinations: (A) Desolvation for the substrate side chain and van der Waals, (B) Desolvation of the substrate side chain and electrostatics, and (C) Site 0 and site -2, as functions of the Z-score (see Methods). (TIF) [file pcbi.1009567.s014.tif]

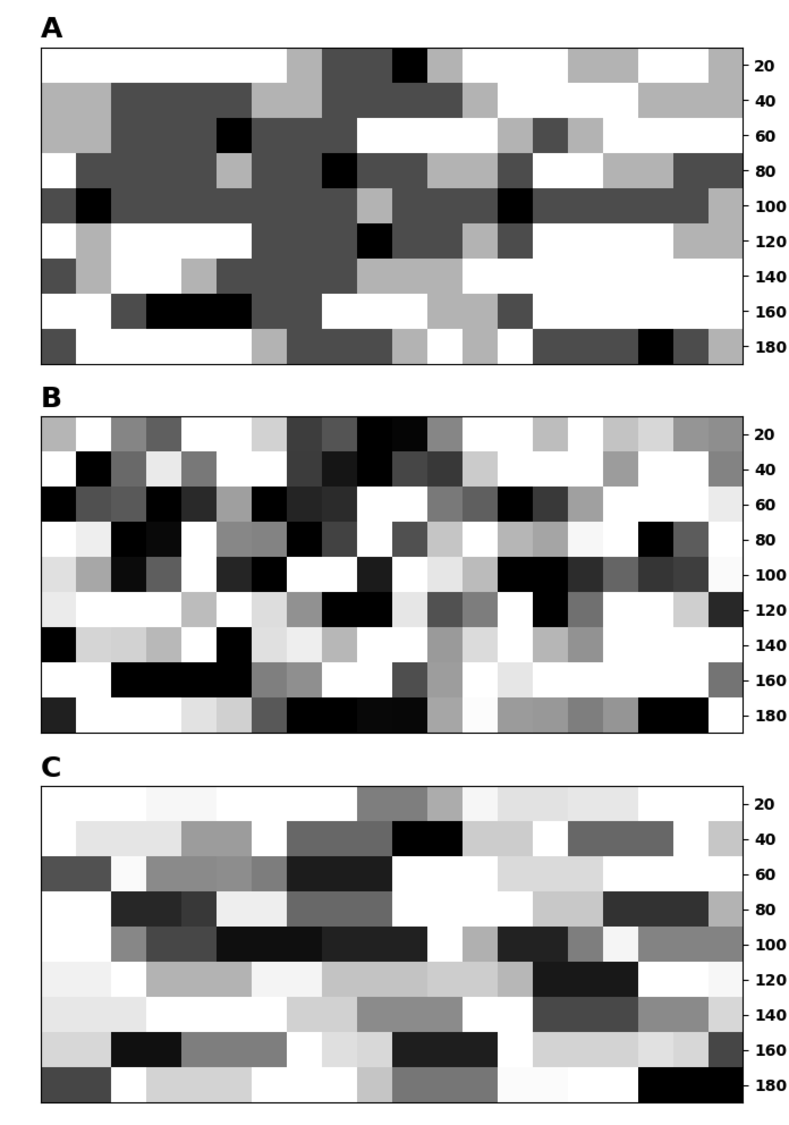

Supplement: S8 Fig — (A) The raw peptide array data from Rudiger, et al. [15]. (B) Predicted peptide array from Rudiger, et al. model. Scores were min-max normalized with thresholds of -3 and 6 in order to clarify the distribution. (C) Predicted peptide array from Paladin model. Min scores for each 13-mer were min-max normalized with thresholds of -10 and -6 to clarify the distribution. Refer to the Methods section in the main text or original publication [15] for details about peptide arrays. (TIF) [file pcbi.1009567.s015.tif]

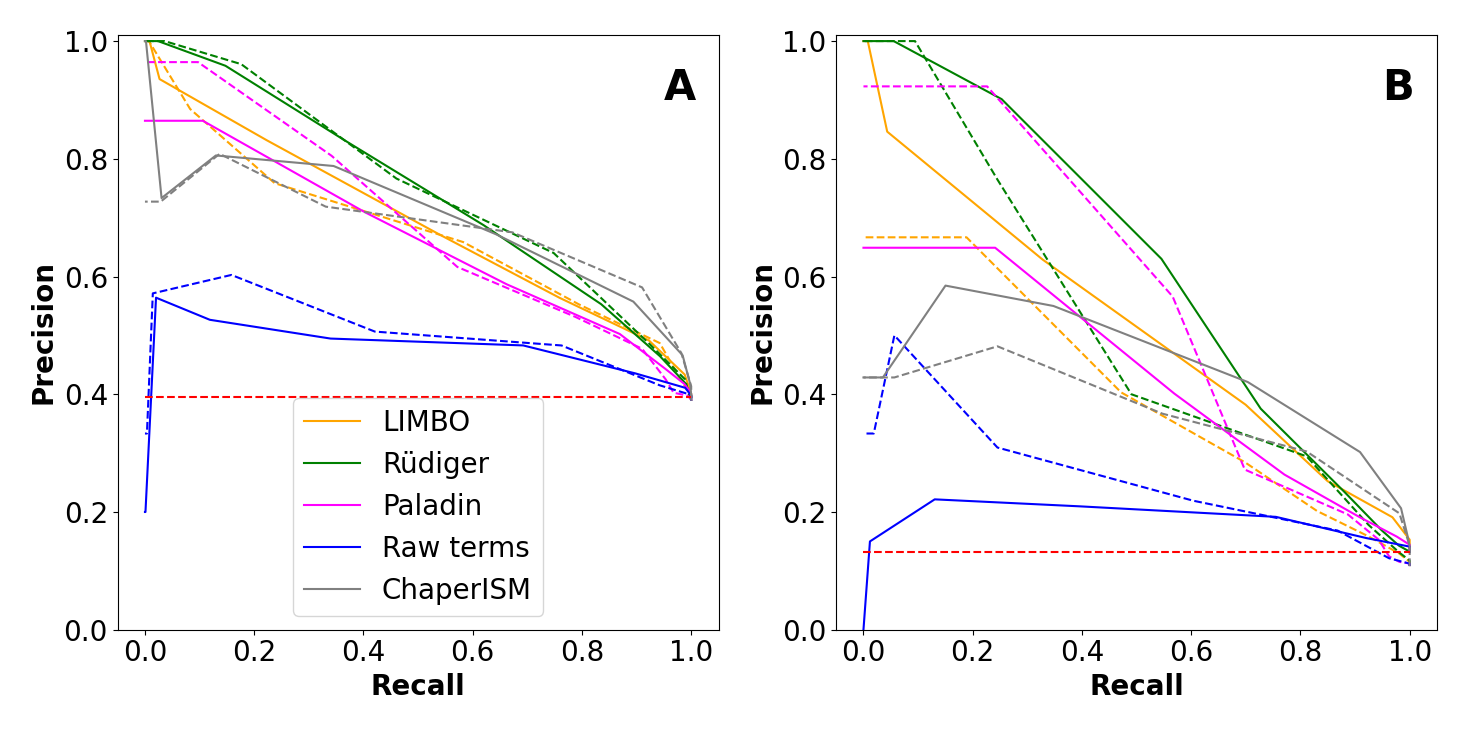

Supplement: S9 Fig — The plots are divided in the same manner as Fig 5 in the main text, with (A) comparing all binders and non-binders and (B) comparing only strong binders and non-binders. The solid lines represent the training set, the solid lines represent test set. The dashed red line at is the fraction of true positives (all binders or strong binders) in the data and reflects the precision of a random predictor. The corresponding areas under the curves (AUC) are given in S4 Table. (TIF) [file pcbi.1009567.s016.tif]

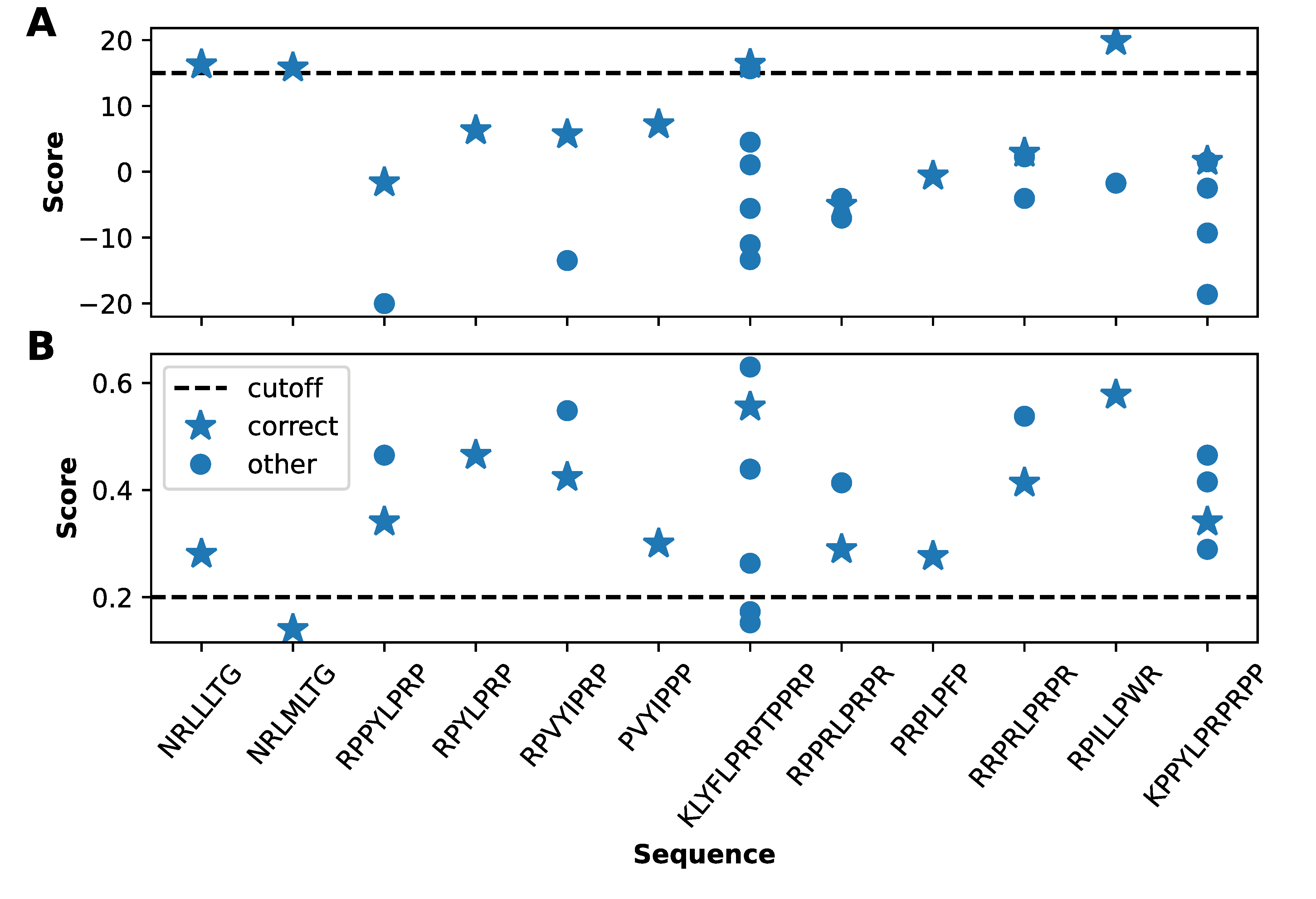

Supplement: S10 Fig — Forward-orientation register predictions for (A) Limbo [16] and (B) ChaperISM [21], which both score 7-mers in only the forward (N- to C-). As in Fig 6 in the main text, stars denote when a 7-mer is “correct”, that is when it is the 7-mer bound in the crystal structure, whereas circles denote all other 7-mers. Both of these algorithms score 7-mers that bind tightly with a higher score (opposite from Paladin’s energy-like score). (TIF) [file pcbi.1009567.s017.tif]

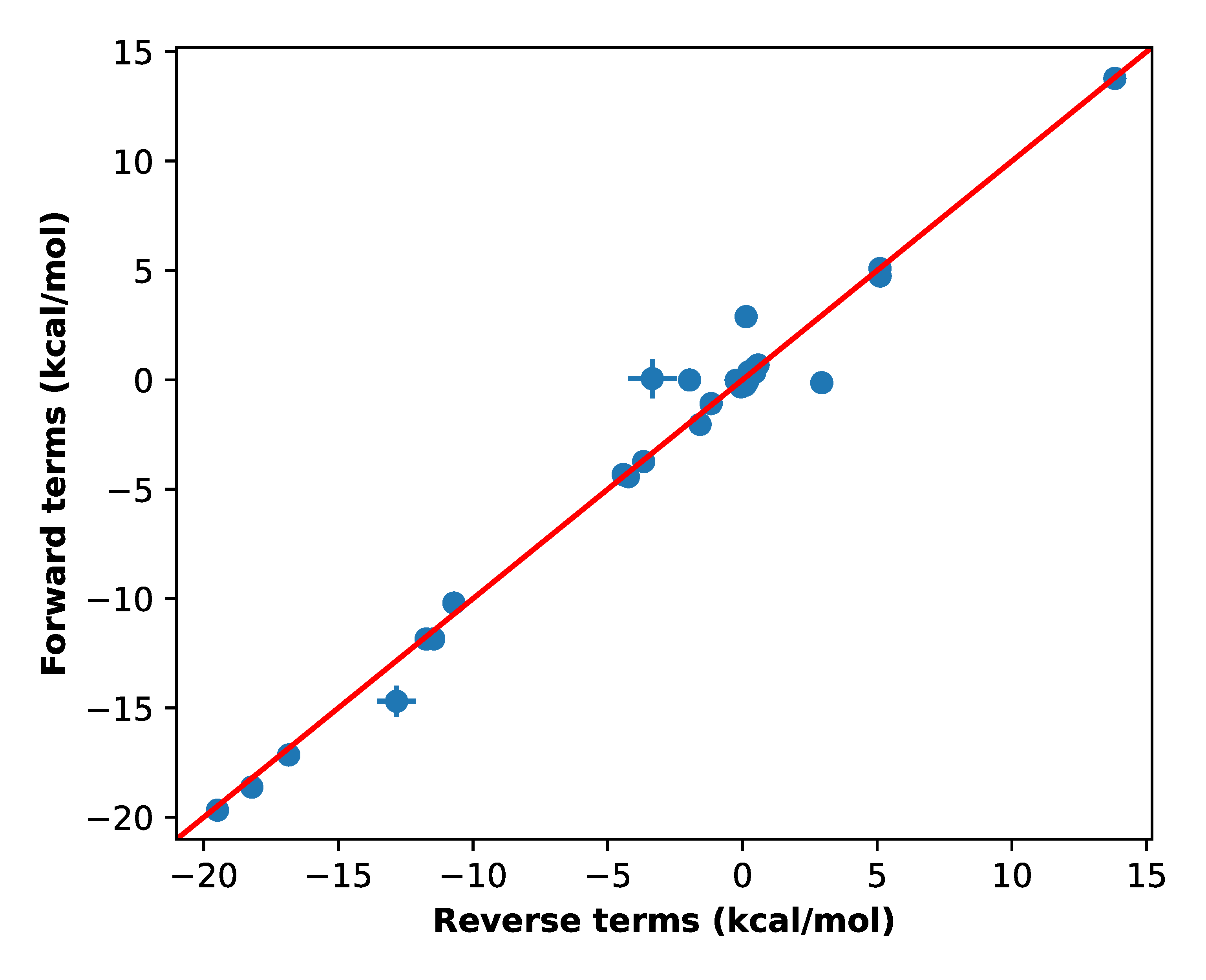

Supplement: S11 Fig — To calculate the vdW, electrostatic and backbone conformational propensity energy terms at site 0, we performed the identical simulation procedure outlined in Methods section of the main text, except we used the backbone position in 4EZY, (NRLILTG bound C- to N-). We simulated the side chains of: A, L, I, Y, W, N, E, K, and R. Error bars are calculated as standard error of the mean. (TIF) [file pcbi.1009567.s018.tif]

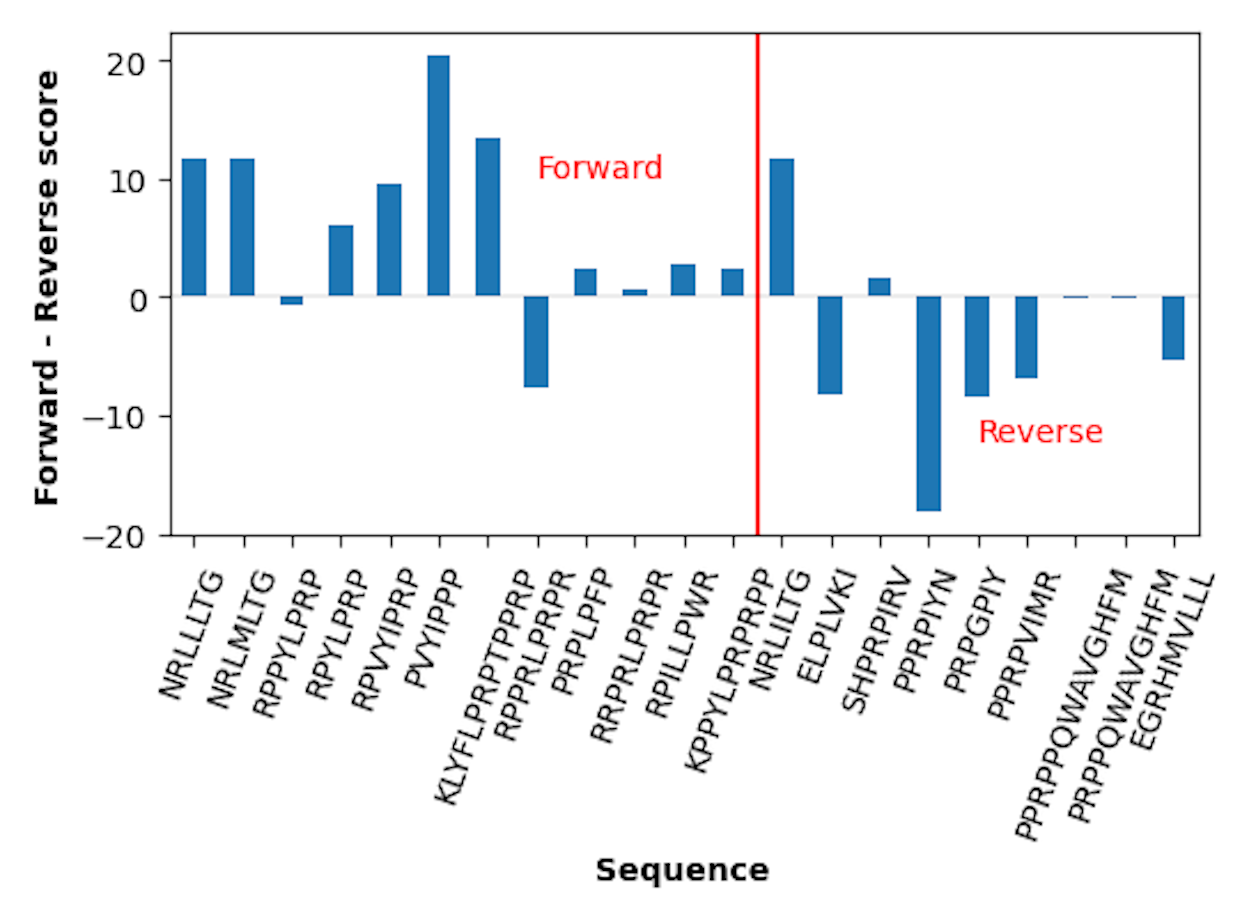

Supplement: S12 Fig — Negative delta (forward–reverse scores) indicates that the reverse orientation is predicted to be preferred, and positive delta for the forward orientation. LIMBO scores peptides of length 7 and uses a higher number to indicate higher affinity to DnaK [13]. (TIF) [file pcbi.1009567.s019.tif]

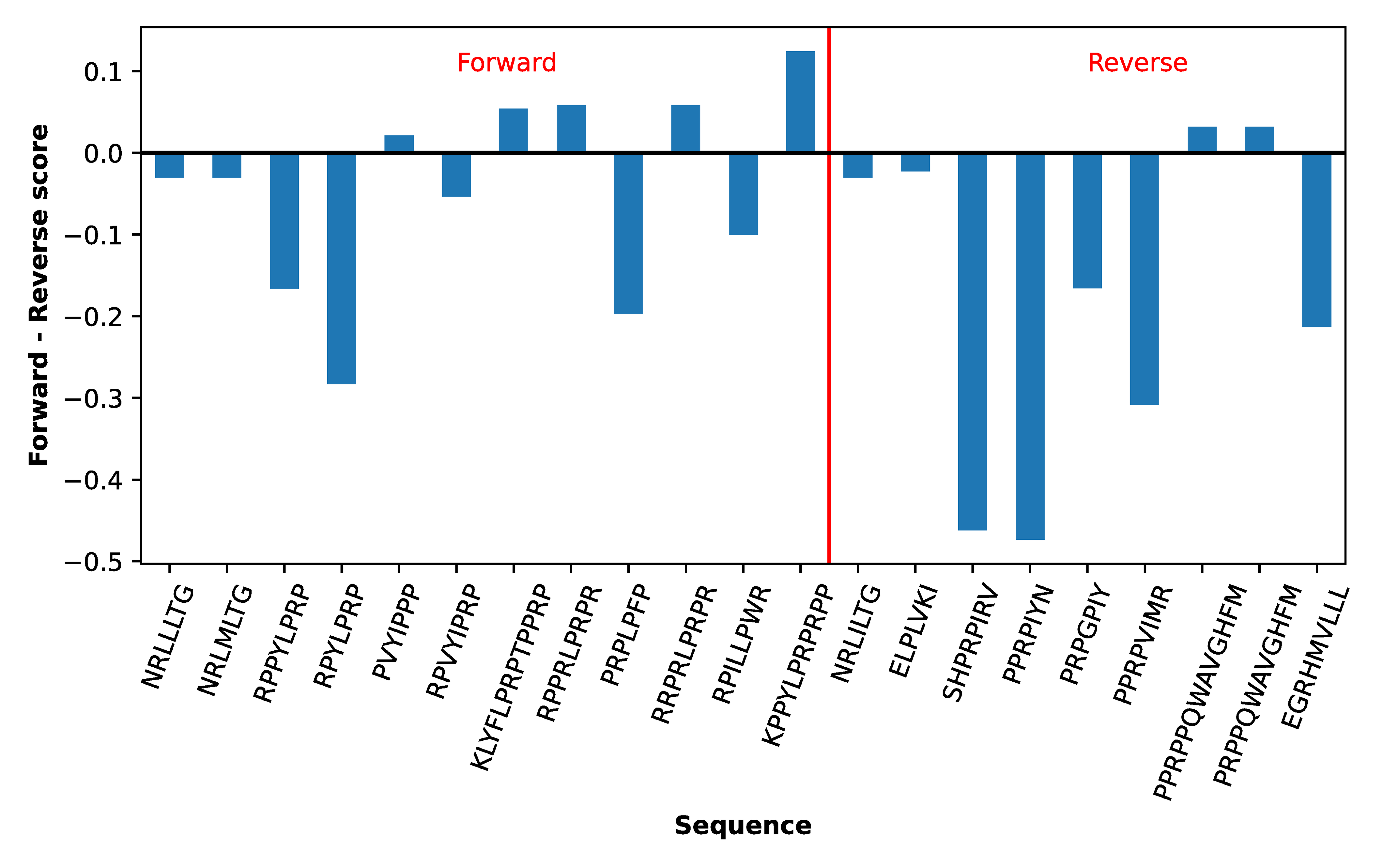

Supplement: S13 Fig — Negative delta (forward–reverse scores) indicates that the reverse orientation is predicted to be preferred, and positive delta for the forward orientation. BiPPred scores peptides 0 to 1, where a higher number indicates higher affinity to the endoplasmic Hsp70 BiP [26]. (TIF) [file pcbi.1009567.s020.tif]

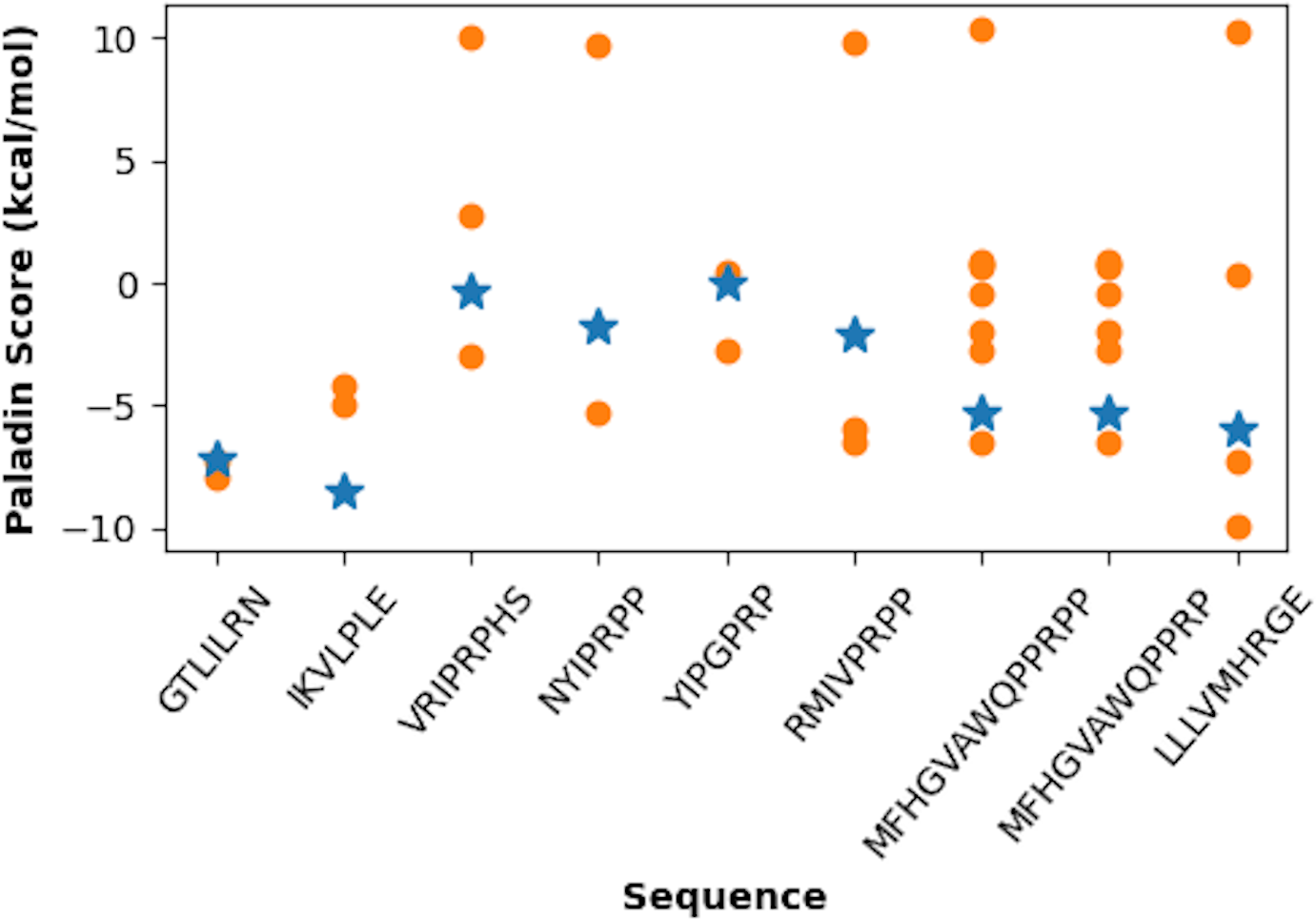

Supplement: S14 Fig — As in Fig 6 in the main text, the Paladin scores are shown for each substrate peptide and all possible binding registries that allow all five sites on DnaK occupied. Both backbone orientations are considered and denoted by color (forward: blue; reverse: orange). The registries as observed in the PDB structures are shown with orange stars. (TIF) [file pcbi.1009567.s021.tif]

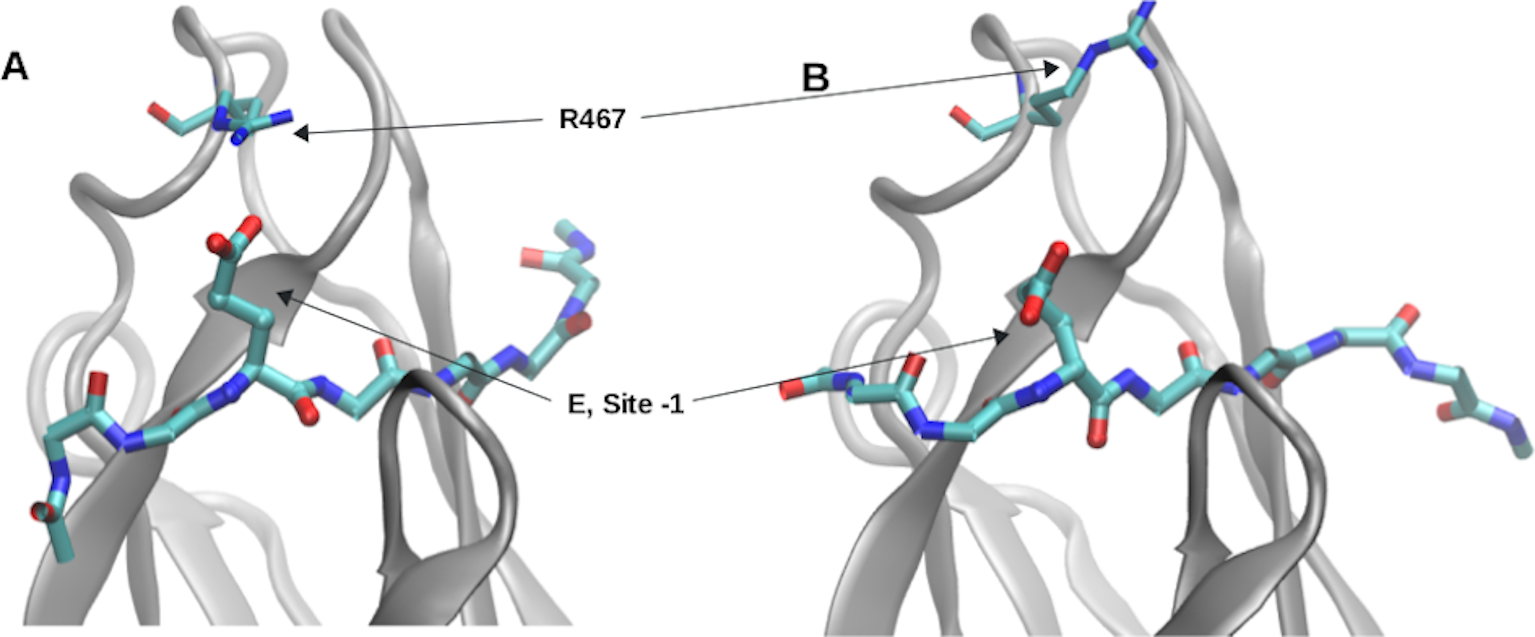

Supplement: S15 Fig — (A) R467 without a salt-bridge (D520-R467) is easily able to interact with E at site -1 of the substrate, resulting in considerable electrostatic contributions that were unexpected. (B) Restrained to mimic effect of salt bridge with lid. Interactions are more as expected (and as reported in parameters for site -1). (TIF) [file pcbi.1009567.s022.tif]

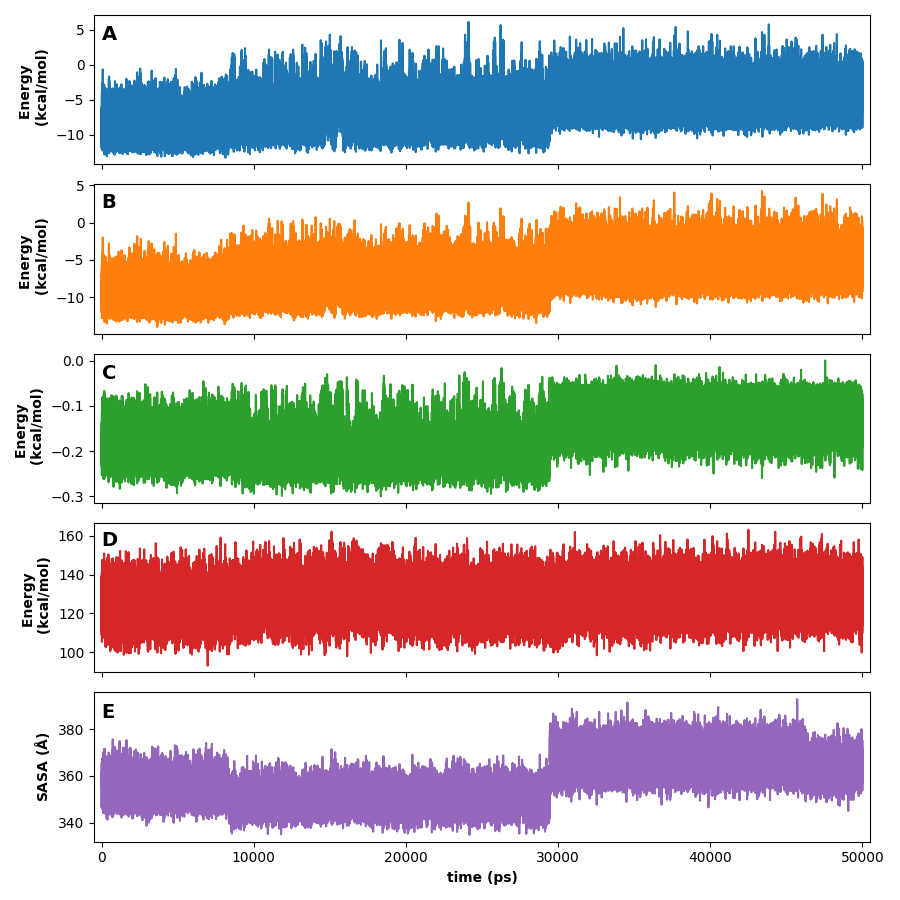

Supplement: S16 Fig — To demonstrate that the length of simulations is appropriate, here is an example of traces for a simulation run for L at site 0 that was 50 ns long, roughly 10 times the length we thought would be necessary. The interactions plotted are (A) Total Interaction Potential energy (kcal/mol), (B) Van der Waals, (C) Electrostatic, (D) Harmonic restraint, and (E) Solvent Accessible Surface Area (Å2). The slight transition just before 30 ns corresponded to a conformational change from the rotamer basin corresponding to the initial configuration into a secondary conformational basin. In fact, all of the short simulations of leucine (5 total, started from 5 rotamers) ended up in similar regions to these to rotamer basins. (TIF) [file pcbi.1009567.s023.tif]

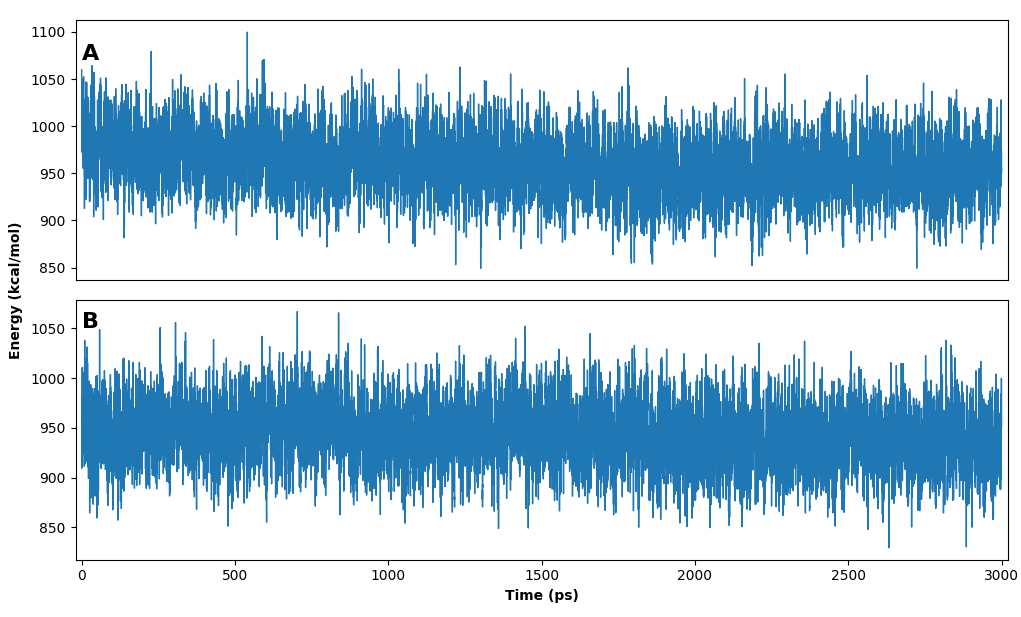

Supplement: S17 Fig — The lowest potential energy traces at site 0 for (A) L and (B) R, respectively. The average total energy was calculated for the last 3/5 of the 3 ns dynamics for each of rotamer-initiated simulation. The lowest-energy trajectory was used to calculate the interaction energies for each site. (TIF) [file pcbi.1009567.s024.tif]
