# Supplementary material for: Physics-based modeling provides predictive understanding of selectively promiscuous substrate binding by Hsp70 chaperones
Source: PLoS Comput Biol. 2021 Nov 4;17(11):e1009567. doi: 10.1371/journal.pcbi.1009567 (PMC8604352; doi:10.1371/journal.pcbi.1009567)
Supplement: Attachment — Submitted filename: Response2Reviewer.pdf [file pcbi.1009567.s025.pdf]

Reviewer #1:

Summary:
Here, Nordquist et al. are interested in develop a Hsp70 peptide binding prediction method based on physics-based simulations to identify client proteins that can bind to SBD region. In their method, they were also able to identify the orientation of the peptides inside the cleft (N-C – "forward"; C-N - "reverse"). The authors also want to provide molecular details of the client protein-SBD binding. The research question is sound and relevant to the field.
Current predictors failed to show molecular details on positions occupied in the SBD or the orientation of the bound peptide. Here, the authors try to fill this gap using their approach. In terms of predicting binding, Paladin performs similar (but still worst according to AUC ROC curve values) to other methods. One positive effect from its training models, possible through transfer learning mechanisms, was the ability to predict the orientation of the peptides (forward vs reverse) in the SBD cleft with accuracy around 70%.
In my opinion, Paladin come to contribute with the field as a new Hsp70 binding predictor, and the main novelty is its capacity to differentiate to some extent the orientation of the peptides, something that is not performed by current predictors.
In light of these facts and the comments below, I recommend the acceptance of this paper with minor revisions.

Abstract and introduction:
Strengths:
- The authors summarize the main research question and key findings.
- The authors covered current literature on the field and explained how previous findings relate to their study.
Weaknesses: - None

Results and conclusions:
Strengths:
- The results support the conclusions.
- Limitations are discussed.

Response: We would like to sincerely thank the reviewer for the support and constructive comments.

Weaknesses:
- Figure 2B is not sufficient to support this statement on Page 8, line 145-147. Here, I suggest to compare these two orientations with a different approach (e.g. peptide residue-SBD distance measures).

Response: We have created **a new Supplementary Movie S1** to more clearly show the conserved backbone and side chain interactions in forward and backward orientations. We have also **modified Figure S1** to include the RMSD values of the βSBD for all complexes with both forward and reverse substrate orientations, which show that βSBD conformations are conserved regardless of the substrate binding orientation. We have further revised the manuscript to clarify: "*The backbone hydrogen bonds between βSBD and the substrate are shown in magenta dashed lines.*

Note that backbone hydrogen bonds involving M404, S427 and A429 completely overlap in two orientations." (**Figure 2 caption)** and *"... reveals that the backbone conformations remain conserved and all five hydrogen bonding interactions between the substrate backbone and βSBD are formed in both orientations (thick and thin magenta dashed lines). Importantly, the highly conserved backbone conformation projects the side chains into identical binding pockets regardless of the orientation. For example, Figure 2B illustrates that the slight shift of Cβ positions does not affect the placement of the central L into site 0. As such, ...*" (**Page 8, lines 168-174**)

- In section "Binding site geometry and surface properties determine the DnaK-substrate selectivit", the authors mention predictions from LIMBO and Rudiger, but not ChaperISM.

Response: ChaperISM uses a position Independent Scoring Matrix (PISM), so doesn't contain information about the relative preferences for individual amino acids at different sites. There was a position-specific scoring matrix mentioned in the original publication, but the better-performing standard options are only for the PISM in (higher performance, default) "qualitative" mode and (slightly lower performance, not used in our paper) "quantitative mode".

- On Page 11, line 233, there is no graph or values showing correlation between vdW interaction energies and size of the side chain, only visual inspection of Figure 4 and S4.

Response: To alleviate the apparent need for such a figure, we have referred to the correlation as "rough" to try to make it clearer that the trend isn't critical to our argument (**Page 12, line 318**), but that the terms as calculated are consistent of what we'd expect. Given that the sidechains are in similar environments, it makes sense that as the number of atoms increases, the number of vdW contacts and thus vdW energy should roughly increase.

- On Page 12, line 235-237, the authors should comment on the possible impact of the length of the simulation (3ns) on the backbone strain term.

Response: Given the highly conserved backbone structures of the complex (e.g., see **Figure 2**), we do not anticipate large scale conformational re-arrangements in response to different substrate side chain groups. Analysis of the simulation trajectories (up to 50 ns) suggested that local structural relaxation occurred rapidly, and all energetics quickly stabilized within 3 ns (e.g., see **Figure S16 and S17**). The convergence is further discussed in Methods (see **Page 23-24, lines 698-703**).

- On Page 15, line 303-305, authors should give details on the data set balance. If the data set is imbalanced, it may be more interesting to show a precision-recall curve. In case the data set is balanced, it would be interesting to show how Paladin would perform in a imbalanced idependent data set (look CD data set at doi:10.1002/prot.25084). Despite this data set came from a different protein, the authors claim that their method can be expanded to other Hsp70 chaperones.

Response: We thank the reviewer for the excellent suggestion. The current dataset is imbalanced, and we have described the distribution of the four sub-categories in the manuscript (**Page 25, lines 773-775**). We have also included **a new SI Figure S9** to compare the precision-recall curves, which supports that Paladin is comparable to other methods in recapitulating the peptide array

data. Additional discussion has been added to the revised manuscript on the new analysis (**Page 16, lines 426-435**). Our current model cannot be extended to other Hsp70s without resampling energy terms with the structure of the new Hsp70, which we respectfully argue is beyond the scope of our current study.

- The AUC ROC values for Paladin, in comparison with Rudiger and ChaperISM, performs worst. The authors have provided possible reasons why this is happening, saying "...the small differences in ROC curves of Paladin and previous models are likely insignificant.". However, none of these reasons were tested.

Response: The criticism is well taken. Indeed, Paladin is the worst in reproducing the peptide array data but the difference is small. In particularly, The AUC PR values are similar for Paladin, ChaperISM and Limbo for all binders (**Table S4, Figure S9**) and Paladin yields the best AUC PR value (0.58) among all four methods evaluated for strong binders (0.37-0.52). In the revised manuscript, we delete the statement noted by the reviewer. Instead, we provide the following discussion: "*On the other hand, the other models including Paladin perform comparably and the differences in ROC/PR curves are small. Importantly, apparent better fitting to the data do not necessarily reflect a superior model. Instead, by incorporating the physics of molecular interactions and using only a small number of free parameters (Table 1), the Paladin model is designed to be capable of predicting key molecular details of binding including backbone orientation and residue registry. This provides an opportunity to reveal the balance of various interactions from different sites in binding specificity and to be extended to modeling other Hsp70s without extensive peptide array data*." (**Page 16-17, lines 465-531**).

- On Page 20, line 417-418, the authors should also mention how ChaperISM performs with Pro at site 0.

Response: Because ChaperISM is position-independent, this cannot be done. ChaperISM scores a P at any position the same. For reference, P has a negative score in ChaperISM (higher score means stronger binder) so P isn't predicted to be favorable at any site.

- The final PSSM matrices with energetic terms need to be included, as this is common practice for prediction methods.

Response: Thank you for pointing this out. We have now included the values of PSSM in a **new SI Table S3**.

Figures and tables:
It is of my opinion that the figures should be carefully revised, either in terms of resolution and labels, but specially in terms of legends. I will give some examples here:

- Figure 1: Since ATP and ADP play an important role and it is mentioned in the text and in the legend of the figure, these molecules should be shown in the figure.

Response: This comment is well-taken, since we do mention the role of ATP/ADP in the functional cycle of DnaK and show the NBD without showing either the molecules themselves or at least the binding sites.

- Figure 2: The interpretation of image B is difficult. I'd recommend (i) different colors for each one of the orientation modes or (ii) two separate images, one with the forward orientation and another one with the reverse orientation.

Response: Thank you for these excellent suggestions to improve the figure. We have considered both of them previously while workshopping this figure extensively over the last year prior to submission. The problems with these suggestions are that: i) Different colors erase the identity of the substrate atoms, which is really crucial detail, and ii) separate images make it difficult to see the striking similarities and symmetric differences between both orientations which are evident in the overlap. Instead, we have now included **a new Supplementary Movie S1** to more clearly show the conserved backbone and side chain interactions in forward and backward orientations.

- Figure 3: There is room for improvement in this image, specially in respect to the surfaces and the representation of the peptides (e.g. ribbon vs stick; colors; transparency).

Response: We agree with the challenge of illustrating these shallow binding surfaces in static 2D figures. This again is a figure that we have revised extensively over time and there seems to be no perfect combination of angle, rendering and coloring. Instead, we have now included **a new Supplementary Movie S2** to more clearly show the site 0 binding pocket.

- Figure 4: I suggest to increase the space in between the charts. Also, the legend is interfering with the data in some cases (e.g. Backbone Conformational Propensity). I suggest to move the legend to another place. Additionally, what FE stands for? This should be written in the legend.

Response: We have expanded FE to Free Energy, fixed the transparency of the legends and adjusted the spacing slightly to minimize clashes.

- Figure S2: Label the residues in the figure. This is a important figure, however, the representation and labels are not adequate to show the relevant point.

- Figure S3: Label the residues in the figure.

Response: Both figures have been updated to include the labels.

- Figure S4: The graph for "Backbone Conformational Propensity" is the same in A-D. Is this correct? If yes, why is so different from site 0?

Response: Thanks for catching the error. The main text Figure 4 is correct, Figure S4 was a mistake we forgot to update during preparation.

- Figure S7: Correct axis labels.

- Figure S8: The legend of the figure do not explain what the star and the spheres represent. "Correct" and "other" may not be the better label, unless explained in the legend.

Response: Thanks, the figure caption has been clarified to explain the use of stars and circles.

- Figure S12: Label the residues in the figure.

- Figure S13: Please, correct y-axis titles appropriately (e.g. type of energy and unit).

- Figure S14: Correct Y-axis (missing parenthesis).

- Table 2: table caption is incomplete.

- Table S1: Include a footnote to explain "Z" amino acid corresponds to cyclohexyalanine.

Response: All figures and tables have been updated as suggested. We greatly appreciate the reviewer's thorough reading of both the main text and SI.

Methods:
Strengths:
- The code and additional files are freely available through GitHub.
- Information about the atomistic simulations are complete.
- Parameterization of Paladin model is explained thoroughly.

Response: Thank you for your positive constructive feedback.

Weaknesses:
- The authors should include:

- a section showing how the structure conservancy was assessed (this is in respect to the first result).

Response: This section has been included. (**Page 23, lines 741-746**)

- one section explaining how the comparison among methods (limbo, rudiger, chaperism, etc) was performed. Also, explain the distribution of the data on the used datasets (e.g. balanced vs imbalanced).

Response: This section has also been added, and a description of the balance of the peptide array dataset has been included. (**Pages 25-26, lines 832-847**)

- README file on github should be updated with more information, specially about the output scores.

Response: Thanks for paying attention to this detail. We have updated the README file to provide more information relevant to an end user.

Minor Issues:
- Introduction is well-written. Results, however, should pass through some language editing to improve clarity.

Response: The revised manuscript has been re-edited as suggested.

- Standardize the nomenclature of amino acids (i.e. one letter vs three letter code).

Response: All references to amino acids are now 1 letter code throughout.

- Page 7, line 112: Please check if reference 25 is the correct one for this sentence.

Response: The reference was intended to point to Rubenstein, et al. 2017 ("MFPred"), thanks for catching this. The in-text references should refer to the proper papers now.

- Page 8, line 154: Please, correct the "Figure 3" statement.

Response: This has been fixed.

- Page 12, line 249: Misplaced reference (ChaperISM reference).

Response: The reference has been added.

- Page 16, line 332: "Figure 5" should be replaced by "Figure 6".

Response: We intended to refer to the ROC figure (Fig 5), this is a reference to ChaperISM performing best on this metric which is most relevant for scanning a bunch of proteins for hotspots.

- Page 16-17, lines 338-341: Repeated text about ChaperISM.

Response: Thanks, the extra text has been removed.

- Page 19, line 395: In Figure 7 I counted 18/22, and not 16/22. Which one is the correct?

Response: Thanks for this insightful question. In fact, we did mean 16/22 here. Even though there are in principle 18 cases with the correct sign, 2 of those have very small free energy differences (ddG < kT or 0.6 kcal/mol). They are considered ambiguous instead of correctly predicted, even though we haven't enough data to be confident of our error we believe the error of Paladin is certainly more than kT. We have revised the manuscript to include a clarification on this point (**Page 20, lines 633-637**).

Reviewer #2:

Summary

In this work, the authors develop a physics-based algorithm to predict binding sites of DnaK, the Hsp70 from E. coli. They posit their algorithm is unique in comparison to existing tools in that it is able to predict orientation (Forward versus reverse) as well as registry (i.e. the specific site occupied by each substrate residue in the DnaK substrate-binding-domain).
In their first set of results, the authors perform a qualitative analysis of a set of previously determined DnaK structures by Zahn and colleagues, and discuss which residues are represented in each position in the substrate and what the underlying energetics are. They also cross-reference these data with LIMBO, a comparable DnaK-substrate prediction algorithm.
Next, the authors describe and validate their own prediction algorithm, Paladin. They show that their algorithm performs similarly to existing algorithms in terms of predicting DnaK-binding peptides in a dataset generated by Rüdiger et al. They further show that Paladin can predict registry of forward- binding peptides, and can predict whether a peptide will bind in the forward or the reverse orientation based on a relatively small set of DnaK-substrate structures.

The authors conclude that the algorithm they have designed is a good DnaK substrate predictor, and given the relatively small extent to which the algorithm was parameterized on the experimental data set, they predict the algorithm to be easily transferable to similar systems.

General
The physics-based algorithm described here adequately predicts DnaK-binding sites, and performs comparably to existing tools for the same purpose. Their use of a 5-residue binding site allows the authors to test for specific substrate registry, in which Paladin outperforms some of its competitors. The algorithm also predicts binding orientation reasonably well, although the authors do not compare the performance therein with other predictors. The explicit use of individual force terms allows for an assessment of which forces drive binding of certain peptides, which is not the case for predictors such as the one devised by Rüdiger, but is comparable to LIMBO. Another advantage of the algorithm seems to be its transferability to similar systems, although the authors should test this before claiming it. Furthermore, the authors provide an interesting analysis of how and why DnaK can interact with substrates in two opposite orientations.

Overall, the authors describe a tool that satisfactorily predicts DnaK binding sites, their binding orientation, and in specific cases, their registry. Although it is not entirely novel and does not massively outperform competitors, the authors describe an additional approach to tackling the important question of DnaK-substrate interactions, and yield additional insight into binding orientation and registry.

Response: Thank you kindly for your constructive and thoughtful remarks.

Major remarks

Line 132: in order to determine how conserved the SBD conformation is, the authors align the structures from Table S1 and determine the RMSD to be small. However, they mention that they only look at structures with substrates in the forward orientation AND structures in which the substate does not contain Pro. Does this mean they have omitted 13 structures out of the 18 forward-oriented structures? In that case, this analysis hardly shows the SBD structure to be highly conserved. Also, does this mean the authors find the SBD needs substantial conformational changes in order to accommodate Pro? This would be interesting information and the authors should comment on this.

Response: The backbone RMSD values of SBD conformations are provided in Figure S1A for all complexes (with and without prolines and regardless of orientation), which are very small (< 2 Å). That is, no substantial conformational change is required for SBD to accommodate different peptide substrates, including proline-containing ones. We note that the presence of proline residues does lead to substantial local movement of the substrate backbone (~1-2 Å). We have revised the manuscript to make this clearer (**Page 7, lines 141-144**).

Line 146: The authors mention that the SBD conformation is highly conserved between forward- and reverse-oriented substrates, though they never test for this since they don't use reverse-oriented substrates in their RMSD analysis. It would be useful to include such an analysis to show that not only the substrate sidechains are located in roughly the same positions, but also the SBD remains in largely the same conformation.

Response: We have **updated Figure S1A** to include the backbone RMSD values of SBDs of all crystal structures, regardless of the substrate orientation. There is no correlation between orientation of the substrate and binding configuration of the SBD. We have also emphasized the more important observation (for our recycling of forward-orientation energy terms) that the site 0 sidechain projects into similar sites regardless of orientation.

In their construction of a physics-based model, the authors consider 6 physical forces, but don't mention hydrogen bond formation at all. Could the authors comment on why they omit a crucial force in protein-protein interaction from their model, and why they feel this choice is justified?

Response: We fully agree that hydrogen bonding is a crucial kind of interaction. Modern general-purpose empirical protein force fields (including CHARMM22, used in this work) don't explicitly include a hydrogen bonding term. Instead, hydrogen bonding is implicitly described using electrostatic and van de Waals interactions. This is in contrast to hybrid empirical/statistical energy functions such as FoldX or Rosetta, which often do contain such terms explicitly. Therefore, there is no omission of critical hydrogen bonding effects in this work.

Line 242-249: How do the authors reconcile the fact that they see only a minor preference for Lysine and no preference for Arg whatsoever, although preferences for both basic references have been described in literature, including by Rudiger et al, and that Arg is represented in many DnaK-substrate interaction structures?

Response: There are two main sources for this apparent limitation of Paladin, namely the RDIE electrostatic model and SASA-based solvation term. In particular, the current model uses the

solvation free energy of each complete residue, whereas residues such as Arg and Lys might more accurately be treated as a hydrophobic sidechain and polar head. We did not attempt to treat these sidechains in this fashion in the final model, mainly due to lack of experimental data on such decomposition and concern of overfitting the peptide array data. For more discussion about the limitation of the RDIE model, please see **Response to Reviewer #3, comment 2a.** We have more strongly emphasized these limitations where appropriate in the manuscript (e.g., **page 17-18, lines 556-580 and page 24, lines 795-797**).

The authors compare Paladin to LIMBO for registry prediction, and find that LIMBO performs similarly to Paladin. However, they never test whether LIMBO is also capable of testing for orientation, and simply show results for their own algorithm. It would be interesting to see if Paladin is uniquely capable of registry prediction, or whether other tools can also do this.
The authors claim the minimal training would allow Paladin to be easily transferable to similar systems. To show this, it should be straightforward to use the experimental data used for the production of the BiPPred tool, and test whether a paladin-type PSSM would also perform well in this system (without retraining on the experimental data, and only using structural information and the weights determined for this algo).

Response: We now have a corresponding orientation prediction for Limbo, shown in **new Figure S12** and briefly discuss it on **Page 20, lines 637-640**.

Extending Paladin to similar systems will require re-calculation of the matrix of interaction terms using physics-based simulations. It does not in principle requires new peptide array data to training the interaction matrix itself, with the assumption that the site and energy term weights are likely similar for all Hsp70s. Nonetheless, it requires substantial amount of new simulations to extend Paladin to BiP and we respectfully argue that it is beyond the scope of this work.

Minor remarks
Abstract: "the chaperone is specific amino acids can vary considerably", this phrase doesn't make sense.

Response: The sentence mentioned here has been corrected.

Line 48: "Paladin provides a physical basis to understand why and how DnaK binds specific peptides". The how is clear, I.e. the molecular modelling gives some information in the driving forces of binding, although some, like H-bonding, are ignored. The "Why" might be a stretch, since the authors don't directly link the hsp70 binding preferences they observe to substrate protein characteristics that warrant hsp70 interaction.

Response: This point is well taken, and the "why" has been removed.

Line 67: "slow on/off binding" should be changed to "slow on/off" rates

Response: The phrase was changed from binding to rates.

Figure 2 caption: "hydrogen bonds are shown in magenta with matching size to their corresponding substrate". This is a little unclear, I assume the sizes of the hydrogen bond lines are thick/thin depending on whether they are formed with forward/reverse orientation. The differences are hard to make out in the figure

Response: This is a good point. We have revised the **Figure 2 caption** to specify three of the five backbone hydrogen bonds that largely overlap between two orientations. Identifying *particular* hydrogen bonds is unnecessary, since one argument in the figure is that those interactions are largely identical.

Line 154: "Figure 3" should be in brackets

Response: Thanks, this has been fixed.

Line 175: should the "+1" here be "-1"?

Response: Yes, this has been corrected.

Lines 188-199. It would be interesting if the authors address whether charge-charge interactions stabilize the Args that are observed in structures at positions -2 and +2. And if they don't, which interactions do stabilize them? Given the general tendency for hsp70s to bind basic residues, this would be interesting to elaborate upon.

Response: Examinations of various energy terms (summarized in **Figure S4**) suggest that the favorable electrostatic interactions of Arg and Lys at sites -2 and +2 are largely cancelled out by the desolvation penalty. As a result, the final Paladin PSSM (see **new Table S3, Figure S5**) suggests only a minor preference for Lysine and no preference for Arg at site +2. The likely reasons for this apparent limitation of Paladin are provided above in response to Reviewer 2's critique (see **Response to Reviewer #2, "*Line 242-249 …", Pages 8-9 of this response letter***).

Line 225: does "the site" refer here to the DnaK binding site? This is unclear.

Response: The "site" does refer to the binding site. This term is calculated from the change in surface area of everything on the protein in contact with the substrate side chain.

Line 459: R4467 should be R467

Response: This has been corrected.

Line 495-496: "A problem with this approach is that some residue side chains, such as those of Arg and Lys, which are hydrophobic chains with polar head groups." This is not a full sentence.

Response: Thank you for pointing this out. The sentence has been revised to "*A problem with this approach is that like all other residues, R and K are considered as a whole sidechain rather than being split into their hydrophobic chains and polar head groups*".

Also, the authors state this is a problem, but don't offer a solution for it. Would this offer an explanation as to why Arg is not favored in their algorithm, yet has been found in binders in vitro, as well as in DnaK- substrate structures?

Response: Please see **Response to Reviewer #2, critique "*Line 242-249 …*"** above.

Figure S7C: there seems to be a yellow square in the top right hand corner of each of the plots, which it seems is not supposed to be there?

Response: These plots have been cleaned up and the legends (the source of the offending squares) were removed.

Lines 338-340: The authors repeat themselves here, one of these sentences should be deleted.

Response: Thanks for pointing this out. The second sentence has been deleted.

Line 429: "revealS"

Response: The typo has been fixed.

It would be useful for the authors to describe how Paladin could be used on sequences of entire proteins. In this work, they design the algorithm and train it on existing experimental data. However, the power of such predictors lies in determining putative binding sites that have not been experimentally validated. Is Paladin capable of screening entire proteins and even proteomes for putative binding sites, and how (e.g. a sliding window approach)? And can it be used to that end by users in its current form?

Response: The model can be evaluated from the command line as a python script. It takes an input file which can have as many FASTA-format sequences as desired. Every 5-mer in the input sequence(s) are scored. The Rüdiger dataset can be scored in about a second. A similar description has been added to the Methods section (**Page 25, lines 827-830)**. In our opinion, many tools exist which can provide equal or better ability to scan for sticky hotspots. If you want a balance of good predictions of binders and non-binders, one can't easily outperform the original Rüdiger algorithm. The power of the Paladin model is in looking deeper at atomistic interactions at each site and comparing them to predict why a substrate binds in some orientation or at some register, or how a change will affect those properties.

Reviewer #3:

Nordquist et al provide a predictor for binding sites of the bacterial Hsp70 chaperone, DnaK, based on molecular dynamics simulations. Hsp70 chaperones are key for the cellular protein folding machinery. The bacterial DnaK homologue is the best understood paradigm for the overall mechanism of action of this family. Key to understand its function in protein folding is to understand how it recognises its substrates. Prediction of such sites is possible since 1997, based on empirical analysis of peptide sequences binding to DnaK.
The present study has a different approach, as it bases the prediction on the analysis of the substrate binding site of DnaK. This conserved Hsp70 binding sites comprises binding positions for five consecutive residues. Contacts are made by both the side chains and the backbone. Additionally, coulomb interactions outside the binding pocket contribute to affinity. The authors test their model against the original data base from the 1997 study.
Their prediction is slightly less accurate than the 1997 algorithm. However, there are two main selling points: (I) The predictor starts from analysing the chemical properties of the chaperone binding site, not the substrate. This may result in more insights into how substrate recognition of Hsp70 works. (II) The predictor can predict the orientation of the peptide stretch in the Hsp70 binding pocket. Thus, if sufficiently accurate this new predictor is an interesting new tool to understand Hsp70 substrate interactions. There are some concerns the authors need to address before publication.

Concerns and advice
1. The predictor does not make full use of the information in the available experimental material.
a. It neglects the flanking regions outside the 5-residue binding site. These regions can contribute via coulomb interactions, and considering their contribution improves the prediction in the Rüdiger algorithm. Thus, taking this into account would improve the quality of prediction.

Response: We thank the reviewer for pointing out the potential contributions of the flanking region and completely agree that their inclusion could improve the algorithm. In fact, this is one of the future directions that we are interested in exploring with Paladin-based approaches. We have chosen not to include them in the algorithm at this point. The primary reason is that these regions are significantly less structured than the central five residues, requiring much more extensive sampling time and likely more accurate (and computationally more demanding) treatment of electrostatic interactions (such using GB or PB-based implicit solvent methods). The current model for the binding of the central five residues will provide a solid basis for extending to modeling the effect of flanking loops. We have revised the manuscript to better explain our choice to focus on the central five residues (**Page 11, lines 318-321**)

b. The manuscript states that there is not information in the peptide data about where in a peptide DnaK may bind. This information can in fact be extracted from the peptides binding data by Rüdiger et al, 1997, as overlapping peptides allow to locate the highest affinity segment when comparing neighbouring peptides. It would allow the authors to test whether their algorithm would predict correctly the common core in overlapping peptides. How does it do on this?

Response: We agree that the peptide array data could be used to extract a best binding region, although in our hands there remains substantial ambiguity about site occupancy and it is difficult

to pinpoint the binding registry at the residue level. We have updated the language in our manuscript to more accurately reflect that the information in the peptide array does contain some information about register but with ambiguity (**Page 14, lines 410; page 22, lines 710-711**).

We have included **two new supplemental figures** which address Paladin's ability to reproduce the peptide arrays. 1) **Figure S8** contains a) the discrete peptide array data, b) the Rudiger model's predictions for comparison, and c) Paladin's predictions. The scores have been normalized to maximize readability. 2) **Figure S9,** which contains Precision-Recall curves, which more faithfully account for classification accuracy and error in this particular dataset (see **Reviewer #2 comment and response starting with "*- The AUC ROC values for Paladin,*"** for more information). The new PR curve and example peptide array predictions provide a similar story that the Rudiger model, as one would expect, fits the peptide array data it was derived from best, and that Paladin does reproduce the peptide array data reasonably well, but like the other predictors is noisy. The ROC and PR metrics provide a succinct and quantitative way to summarize this fitting.

2. Some parameters in the prediction matrix do not fit previous experimental data.

a. The negative effect of acidic residues in the 5-residue binding site only has a moderate effect, while positive charges are dramatically disfavoured. This is contrast to both the statistical composition of the binding peptides and the finding that negatively charged residues wipe out binding, while positively charged residues do not. This suggests that the MD analysis of the present study does not adequately takes charges into account.

Response: We thank the reviewer for the insightful comment. We agree that the current Paladin model has apparent limitations in recapitulating the binding preference of charged residues, despite the apparent overall success. This may be attributed to two key approximations, namely RDIE electrostatics and SASA solvation model. Please refer to **our response above to Reviewer #2, critique "*Line 242-249: …* " (pages 8-9 of this response letter)** for additional details. In addition, we note that these approximations are adopted not only for computational efficiency, but also to allow full pair-wise decompositions of all energy terms required for deriving a PSSM. For the more accurate generalized Born (GB) model, the effective Born radii depend on the conformation of the whole complex and the interaction energies can not be rigorously decomposed into a sum of side chain/site interaction contributions. We have added this discussion to the Methods section. (**Page 23, lines 757-762)**

b. The structural basis of the predictor is based on the Hendrickson structure of the DnaK substrate binding domain. This study describes that the central position 0 is tailored for Leucine. This is consistent with the analysis of the composition of the residues in this region, which shows Leu strongly favoured over all other residues. This is not the case for the predictor here, and it is unclear why. Here Ile is almost as good as Leu.

Response: We agree with the reviewer that it is somewhat puzzling that our analysis suggests that Leu and Ile would be comparably compatible with site 0. We comment on this in the text with respect to Paladin's inability to identify the flip of orientation between NR**LL**LTG (forward) and NR**L**ILTG (reverse), which keeps L at site 0 in both cases (**Page 21, lines 671-674**). In addition, we note that the complementary experimental work in the Gierasch lab and previous experimental

work has not infrequently found I in the central site. For example, complexes listed in Table S1 include 3 I and 15 L in site 0, which means you'd expect about -0.6*ln(3/15) = 0.97 kcal/mol based on this extremely limited dataset. Nonetheless, the description of nonpolar interactions in our atomistic simulations should be accurate and reliable. The peculiar preference of L over I thus may not be explained by local interactions.

3. The peptide data to analyse forward/reverse binding are based on a relatively small number of peptides, which also appear relatively untypical. The impact of this is not adequately discussed, nor are data taken into account assessing the impact of D-amino acids on DnaK backbone binding (Rüdiger et al, 2001; Feifel et al, 1998).

Response: We have further highlighted the limitations of the reverse binder dataset in the manuscript (**Pages 21, lines 652-653**).

While a fascinating question, considering the effects of D-amino acids is beyond the scope of the current work as our goal was to predict DnaK binding sequences within physiological substrates. The possibility of binding of D-amino acids would become important in design of novel binders, for example as inhibitors. We have added a comment to this effect in the discussion, with citations to the two references the reviewer has noted. (**pages 22-23, lines 728-738**)

4. There are experimental data available on the impact of the hydrophobic arch over the substrate binding cleft on affinity and specificity (Mayer et al, 2000, Rüdiger et al, 2000). These are not taken into account nor adequately discussed.

Response: We thank the reviewer again for pointing out this important aspect of substrate binding to DnaK. We emphasize that the hydrophobic arch is present in all atomistic simulations, and thus all interactions between it and the substrate are included in the interaction matrix. Those residues are mentioned explicitly in our description of the binding sites and in a supplemental figure (**Figure S3**). We have now made **revision to the text** to more clearly point out their importance and cited the papers the reviewer notes (**Page 9, lines 228-230**). It could be an interesting future extension of our analysis to parse the contributions of each site in the beta-subdomain and the contributions of the arch residues, but this is beyond the scope of the current study.

Have the authors made all data and (if applicable) computational code underlying the findings in their manuscript fully available?

Reviewer #1: No: The final PSSM matrices with energetic terms need to be included, as this is common practice for prediction methods.
Reviewer #2: Yes
Reviewer #3: Yes

Response: Thank you for reminding us of this, it has been now included (see **Table S3**).
